# Red cell distribution width (RDW) is independently associated with all-cause mortality in adult patients with osteomyelitis admitted to the intensive care unit

**DOI:** 10.1371/journal.pone.0332211

**Published:** 2025-09-16

**Authors:** Likai Liang, Zhe Chen, Zongyun He, Haibing Tao, Yang Chen, Tao Liu

**Affiliations:** 1 Department of Hand and Foot Surgery, Yiwu Central Hospital, Yiwu, China; 2 Liverpool Centre for Cardiovascular Science at University of Liverpool, Liverpool John Moores University and Liverpool Heart and Chest Hospital, Liverpool, United Kingdom; Ferdowsi University of Mashhad, IRAN, ISLAMIC REPUBLIC OF

## Abstract

**Objective:**

This study aims to investigate the correlation between red cell distribution width (RDW) and overall mortality in adults diagnosed with osteomyelitis.

**Methods:**

In this retrospective study, we examined data from the Medical Information Mart for Intensive Care IV (MIMIC-IV) database, comprising 2,700 patients with osteomyelitis and available RDW data on the initial day of admission. Employing Kaplan-Meier survival analysis, we assessed the incidence rate of primary outcome events among groups categorized by RDW levels (Q1: RDW ≤ 13.5, Q2: 13.5 < RDW ≤ 14.6, Q3: 14.6 < RDW ≤ 16.1, Q4: 16.1 < RDW), with differences evaluated using the Log-rank test. Subsequently, Cox proportional hazards analyses were conducted to investigate the correlation between RDW and the overall mortality risk. Additionally, we performed stratified analyses based on factors such as gender, congestive heart failure, diabetes, and myocardial infarction to scrutinize the consistency of RDW’s prognostic significance.

**Results:**

Over the 90-day follow-up, 10.7% of patients with osteomyelitis succumbed. Unadjusted RDW correlated significantly with in-hospital, 30-day, and 90-day mortality (*p* < 0.05). Higher RDW levels proved more effective in predicting increased risks. RDW emerged as an independent prognostic indicator, showing no significant interactions with sex, congestive heart failure, diabetes, and myocardial infarction (interaction *p*-values: 0.254 to 0.920).

**Conclusions:**

The noteworthy link between RDW and heightened all-cause mortality in patients with osteomyelitis who were hospitalized in the intensive care unit highlights RDW’s potential as a valuable marker for identifying at-risk individuals during hospitalization.

## Introduction

Osteomyelitis, a challenging infectious bone condition, poses difficulties for healthcare professionals and patients alike. The incidence is 21.8 cases per 100,000 person-years in the United States [1]. Osteomyelitis frequently arises as a complication of open fractures, internal fixation procedures, diabetic foot ulcers, or hematogenous bone infections. The primary anatomical locations for the occurrence of osteomyelitis are typically the tibia and femur [2]. In recent years, there has been a noticeable rise in the incidence of vertebral osteomyelitis. The overall mortality rate has been documented as high as 20%, with a notably heightened risk observed in the first year following diagnosis [3,4]. The diagnosis of osteomyelitis mainly depends on clinical manifestation, laboratory tests and imaging studies. In the early stage of the disease, clinical manifestations are often subtle, and imaging shows no obvious signs of infection, making early diagnosis challenging [5]. Inflammatory markers, such as interleukin-6 (IL-6), tumor necrosis factor-alpha (TNF-α), procalcitonin (PCT), and C-reactive protein (CRP), serve as pivotal indicators in assessing the severity of osteomyelitis. These markers underscore the significant impact of inflammation levels on both the diagnostic and prognostic aspects of osteomyelitis [6,7]. Red cell distribution width (RDW), typically expressed as a percentage (%), is a parameter routinely evaluated in standard blood analyses. It serves as an indicator of the variability in the size of erythrocytes, and has long been used in the hematology laboratory to distinguish various types of anemia [8]. In recent years, numerous studies have highlighted the predictive potential of RDW in anticipating the occurrence or prognosis of various medical conditions. These encompass pneumonia, chronic obstructive pulmonary disease, myocardial infarction, stroke, drug-induced liver injury, Hodgkin’s lymphoma, sepsis, fractures, anemia, brain death, cancer and other diseases [9–17]. Remarkably, the significance of RDW is increasingly recognized as a robust and independent predictor of mortality in the general population [18,19]. Perlstein et al. discovered a noteworthy correlation, indicating that a one-standard-deviation (1-SD) increase in RDW is associated with a 23% higher risk of all-cause mortality, a 28% higher risk of mortality due to cancer, and a 32% higher risk of mortality from chronic lower respiratory diseases. Importantly, these associations remained significant even after comprehensive adjustments for factors such as age, sex, race/ethnicity, and other variables [20].

Although RDW is a known prognostic factor in many diseases, its role in patients with osteomyelitis, especially those in the intensive care unit (ICU), has not been well studied. The association between RDW and overall mortality in adult patients with osteomyelitis admitted to the ICU remains to be elucidated. In this study, our objective was to examine the association between RDW and overall mortality, and to assess the influence of RDW on the prognostic outcomes of patients with osteomyelitis admitted to the ICU.

## Methods

### Study population

This study represents a retrospective observational inquiry. Data for analysis was retrieved from an online international database known as the Medical Information Mart for Intensive Care IV (MIMIC-IV). The MIMIC-IV database comprises clinical information related to 454,324 patients admitted to the ICU at Beth Israel Deaconess Medical Center (BIDMC), Boston, USA, between 2008 and 2019. Upon successfully completing an examination and obtaining certification, access to this database is authorized. Author YC obtained the required permissions to access the dataset (Record ID 36328122) and took on the responsibility of extracting the data. The project received approval from the Institutional Review Boards of both the Massachusetts Institute of Technology and BIDMC. Additionally, a waiver for informed consent was granted.

**Fig 1:**
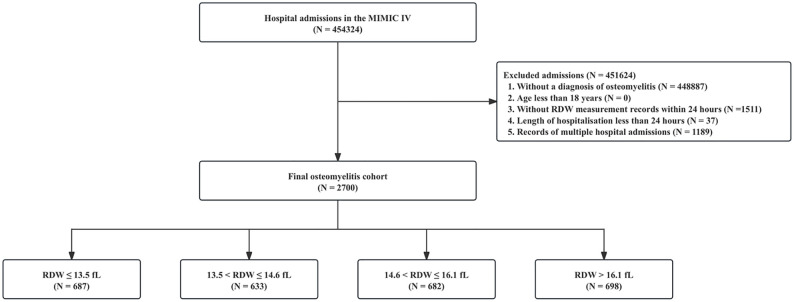
The patient screening flow chart.

We conducted a thorough analysis of data obtained from 5,437 patients (aged 18 years and older) with osteomyelitis (ICD-9code: 730.x; ICD-10: M90.9) who were admitted to MIMIC-IV. Patients without RDW data on the initial day of admission were excluded from the study. Furthermore, our analysis was confined to the initial hospital admission for patients whose hospital stay exceeded 24 hours. Patients with records of multiple hospital admissions were also excluded. The final study cohort consisted of a total of 2,700 patients with osteomyelitis, who were stratified into four groups according to the quartiles of their RDW values on the initial day of admission. The patient screening flow chart is presented in Fig 1.

### Variable extraction

The baseline characteristic data from the initial 24 hours of patients with osteomyelitis was retrieved from the MIMIC-IV database, including age, sex, race, comorbidities, laboratory results, interventions and clinical outcomes. The comorbidities included congestive heart failure (CHF), myocardial infarction (MI), peripheral vascular disease, cerebrovascular disease, chronic pulmonary disease, rheumatic disease, peptic ulcer disease, paraplegia, diabetes, liver disease, cancer and renal disease. Laboratory results included anion gap, sodium, potassium, calcium, creatinine, blood urea nitrogen, bicarbonate, chloride, glucose, hematocrit, red blood cell, mean red blood cell volume, mean corpuscular hemoglobin, white blood cell, platelet and hemoglobin. Interventions included mechanical ventilation, hemodialysis and vasopressor administration. Clinical outcomes included length of hospital stay, in-hospital mortality, 30-day post-admission mortality and 90-day post-admission mortality. If a variable was recorded more than once in the first 24 hours, we used its average value. The original data is presented in S1 Table.

### Outcomes

The primary outcome of this study was in-hospital all-cause mortality. The secondary outcomes included 30-day and 90-day post-admission mortality.

### Statistical analysis

All statistical analyses were performed using R software (version 4.2.1) and SPSS (version 27). A two-sided p-value < 0.05 was considered statistically significant. Group Stratification: Patients were divided into four groups according to the quartiles of RDW values measured on the first day of hospital admission. Quartile cut-off points were derived from the distribution of RDW in the study cohort. This stratification enables the comparison of outcomes across increasing RDW levels. Descriptive Statistics: Baseline characteristics were presented as mean ± standard deviation (SD) or median with interquartile range (IQR) for continuous variables, and as counts with percentages for categorical variables. Comparisons of continuous variables between RDW quartile groups were performed using one-way ANOVA or Kruskal-Wallis test, depending on data normality. Categorical variables were compared using Chi-square test or Fisher’s exact test when appropriate. These tests assess whether baseline characteristics and outcomes vary significantly across RDW levels. Survival Analysis: We used Kaplan–Meier survival curves to visualize survival probabilities across RDW quartile groups. Differences between groups were evaluated using the log-rank test. This non-parametric test determines whether the survival experiences of different groups are statistically different.

Multivariate Cox Proportional Hazards Models: To quantify the association between RDW and all-cause mortality (in-hospital, 30-day, and 90-day), we fitted Cox regression models. Results were expressed as hazard ratios (HRs) with 95% confidence intervals (CIs).

Model 1: Categorized red cell distribution width without adjustment.

Model 2: Model 1 adjusted by age, gender, race.

Model 3: Model 2 additionally adjusted for comorbidities (CHF, MI, peripheral vascular disease, cerebrovascular disease, chronic pulmonary disease, rheumatic disease, peptic ulcer disease, paraplegia, diabetes, liver disease, cancer, renal disease) and interventions (mechanical ventilation, hemodialysis and vasopressor administration).

Subgroup and Interaction Analysis: Stratified Cox regression was performed based on variables including gender, CHF, diabetes, and MI to assess the consistency of RDW’s prognostic value. Interaction terms were included to test for effect modification.

Predictive Performance Evaluation: To assess the discriminatory power of RDW, we constructed Receiver Operating Characteristic (ROC) curves and calculated the Area Under the Curve (AUC) for each mortality outcome. AUC values of 0.7–0.8, 0.8–0.9, and >0.9 indicate acceptable, excellent, and outstanding discrimination, respectively.

Non-linear Relationship Assessment: We applied Restricted Cubic Spline (RCS) regression to model the potential non-linear association between RDW and mortality risk. This method provides a smoothed estimate of the risk relationship and helps detect thresholds or non-linear patterns that linear models may miss.

## Results

### Baseline characteristics and clinical outcomes

The baseline characteristics and clinical outcomes of patients with osteomyelitis are presented in Table 1. A total of 2700 patients were included: 1,748 (64.7%) were female and 952 (35.3%) were male, with a mean age of 72.52 years; 1942 (71.9%) were White, and the median hospital stay was 7.79 days. In-hospital, 30-day and 90-day post-admission mortality rates were 3.9%, 5.3% and 10.7%, respectively. The highest quartile RDW group was older, more often male, and had more comorbidities such as CHF, MI, chronic lung disease, rheumatic disease, and malignancy compared to all other groups. Importantly, the highest quartile RDW group had a longer length of stay, and significantly higher in-hospital, 30-day and 90-day post-admission mortality rates (*p* < 0.001).

**Table 1:** Baseline characteristics and clinical outcomes of patients with osteomyelitis.

Characteristics	All	Q1	Q2	Q3	Q4	*p*-value
**N**	2700	687	633	682	698	
**Age (years)**	63.33 ± 15.03	59.51 ± 15.39	62.59 ± 15.06	65.13 ± 14.76	66.00 ± 14.08	< 0.001; ^***^
**Male, n (%)**	1748 (64.7)	490 (71.3)	416 (65.7)	438 (64.2)	404 (57.9)	< 0.001; ^***^
**Race, n (%)**						0.920
** White**	1942 (71.9)	494 (71.9)	454 (71.1)	497 (72.9)	497 (71.2)	
** Non-white**	758 (28.1)	193 (28.1)	179 (28.3)	185 (27.1)	201 (28.8)	
**Comorbidities, n (%)**						
** Congestive heart failure**	690 (25.6)	72 (10.5)	109 (17.2)	196 (28.7)	313 (44.8)	< 0.001; ^***^
** Myocardial infarction**	326 (12.1)	42 (6.1)	68 (10.7)	96 (14.1)	120 (17.2)	< 0.001; ^***^
** Peripheral vascular disease**	665 (24.6)	146 (21.3)	153 (24.2)	164 (24.0)	202 (28.9)	0.010; ^**^
** Cerebrovascular disease**	194 (7.2)	29 (4.2)	45 (7.1)	55 (8.1)	65 (9.3)	0.002; ^**^
** Chronic pulmonary disease**	471 (17.4)	82 (11.9)	89 (14.1)	138 (20.2)	162 (23.2)	< 0.001; ^***^
** Rheumatic disease**	102 (3.8)	10 (1.5)	18 (2.8)	35 (5.1)	39 (5.6)	< 0.001; ^***^
** Peptic ulcer disease**	41 (1.5)	2 (0.3)	10 (1.6)	10 (1.5)	19 (2.7)	0.003; ^**^
** Paraplegia**	141 (5.2)	15 (2.2)	29 (4.6)	36 (5.3)	61 (8.7)	< 0.001; ^***^
** Diabetes**	1571 (58.2)	399 (58.1)	366 (57.8)	385 (56.5)	421 (60.3)	0.536
** Liver disease**	268 (9.9)	45 (6.6)	57 (9.0)	75 (11.0)	91 (13.0)	< 0.001; ^***^
** Cancer**	134 (5.0)	18 (2.6)	24 (3.8)	39 (5.7)	53 (7.6)	< 0.001; ^***^
** Renal disease**	901 (33.4)	148 (21.5)	172 (27.2)	238 (34.9)	343 (49.1)	< 0.001; ^***^
**Laboratory results**						
** Anion gap (%)**	15.00 (13.00, 17.00)	15.00 (13.00, 17.00)	15.00 (13.00, 17.00)	15.00 (13.00, 17.00)	15.00 (13.00, 18.00)	< 0.001; ^***^
** Sodium (mmol/L)**	138.00 (135.00, 140.00)	138.00 (135.00, 140.00)	138.00 (136.00, 140.00)	138.00 (135.00, 141.00)	138.00 (135.00, 141.00)	0.008; ^**^
** Potassium (mmol/L)**	4.30 (3.90, 4.70)	4.30 (4.00, 4.60)	4.20 (3.90, 4.60)	4.30 (4.00, 4.70)	4.30(3.90, 4.70)	0.102
** Calcium (mg/dL)**	8.90 (8.40, 9.30)	9.00 (8.50,9.40)	8.90 (8.50, 9.40)	8.80 (8.40, 9.30)	8.80 (8.30, 9.30)	< 0.001; ^***^
** Creatinine (mg/dL)**	1.00 (0.80, 1.50)	0.90 (0.80, 1.20)	1.00 (0.80, 1.40)	1.00 (0.80, 1.50)	1.20 (0.90, 2.10)	< 0.001; ^***^
** Blood urea nitrogen (mg/dL)**	20.00 (14.00, 30.00)	17.00 (13.00, 24.00)	19.00 (14.00, 26.00)	20.50 (14.00, 32.00)	24.00 (14.00, 42.00)	< 0.001; ^***^
** Bicarbonate (mmol/L)**	26.00 (23.00, 28.00)	26.00 (24.00, 28.00)	26.00 (24.00, 28.00)	26.00 (24.00, 28.00)	26.00 (24.00, 28.00)	0.261
** Chloride (mmol/L)**	101.00 (98.00, 104.00)	100.00 (97.00, 103.00)	101.00 (98.00,104.00)	101.00 (98.00,104.00)	101.00 (97.00,104.00)	0.004; ^**^
** Glucose (mg/dL)**	124.00 (97.00, 186.00)	131.00 (99.00,205.00)	121.00 (97.00, 190.50)	121.00 (96.00,177.00)	124.00 (94.00, 175.25)	0.007; ^**^
** Hematocrit (g/dL)**	36.30 (32.23, 40.10)	37.70 (34.60, 40.90)	37.00(33.34, 40.70)	35.80 (31.98, 39.60)	34.20 (30.08, 38.50)	< 0.001; ^***^
** Red blood cell (×10** ^**6**^ **/µL)**	4.06±0.66	4.21±0.58	4.13pm 0.64	4.03±0.65	3.89±0.74	< 0.001; ^***^
** Mean red blood cell volume (fL)**	89.00 (85.00, 93.00)	90.00 (86.00, 93.00)	90.00 (86.00, 93.00)	89.00 (84.00, 93.00)	89.00 (84.00, 95.00)	0.023; ^*^
** Mean corpuscular hemoglobin (pg)**	29.80 (28.10, 31.30)	30.30 (29.00, 31.50)	29.90 (28.50, 31.40)	29.45 (27.80, 31.10)	29.00 (26.90, 31.00)	< 0.001; ^***^
** White blood cell (×10** ^**3**^ **/µL)**	8.80 (6.90, 11.80)	9.00 (7.00, 11.70)	9.00 (7.00, 11.45)	8.70 (6.70, 12.10)	8.70 (6.80, 11.90)	0.937
** Platelet (×10** ^**3**^ **/µL)**	258.00 (199.00, 332.00)	266.00 (205.00, 333.00)	261.00(204.00, 332.00)	251.50 (199.00, 326.23)	253.00 (184.75, 338.75)	0.097
** Hemoglobin (g/dL)**	12.10 (10.60, 13.40)	12.80 (11.50, 14.00)	12.50 (11.10, 14.00)	11.50 (10.40, 13.20)	11.10 (9.60, 12.60)	< 0.001; ^***^
**Interventions (1**^**st**^ **24 h), n (%)**						
** Mechanical ventilation**	205 (7.6)	18 (2.6)	38 (6.0)	50 (7.3)	99 (14.2)	< 0.001; ^***^
** Hemodialysis**	192 (7.1)	8 (1.2)	19 (3.0)	46 (6.7)	119 (4.4)	< 0.001; ^***^
** Vasopressor administration**	392 (14.5)	38 (5.5)	62 (9.8)	94 (13.8)	198 (28.4)	< 0.001; ^***^
**Clinical outcomes**						
** Length of hospitalized stay, days**	7.79 (4.92, 12.88)	6.83 (4.25, 10.58)	7.17 (4.79, 11.79)	8.06 (5.13, 13.04)	9.04 (5.58, 17.34)	< 0.001; ^***^
** In-hospital mortality, n (%)**	106 (3.9)	2 (0.3)	12 (1.9)	17 (2.5)	75 (10.7)	< 0.001; ^***^
** 30-day post-admission mortality, n (%)**	144 (5.3)	2 (0.3)	16 (2.5)	28 (4.1)	98 (14.0)	< 0.001; ^***^
** 90-day post-admission mortality, n (%)**	288 (10.7)	12 (1.7)	39 (6.2)	67 (9.8)	170 (24.4)	< 0.001; ^***^

Data are expressed as mean ± standard deviation, median (interquartile range), or number (%). Analysis of variance (or the Kruskal-Wallis test) and Chi-square (or Fisher’s exact) tests were used for comparisons among groups. Statistical significance was set at *p* < 0.05. Red cell distribution width was grouped as follows: Q1: RDW ≤ 13.5, Q2: 13.5 < RDW ≤ 14.6, Q3: 14.6 < RDW ≤ 16.1, Q4: 16.1 < RDW.

* *p* < 0.05;

** *p* < 0.01;

*** *p* < 0.001.

### RDW was an independent risk factor for in-hospital, 30-day post-admission, and 90-day post-admission all-cause mortality

According to Table 2, unadjusted RDW was significantly associated with in-hospital, 30-day post-admission, and 90-day post-admission all-cause mortality. After adjusting for confounders in the multivariate Cox regression, RDW remained associated with in-hospital all-cause mortality (*p-*values less than 0.05), 30-day post-admission all-cause mortality (**p* *< 0.05), and 90-day post-admission all-cause mortality (**p* *<* *0.05).

**Table 2:** Multivariate Cox or logistic regression analyses for categorized RDW and clinical outcomes in patients with osteomyelitis.

Outcomes		Model 1	*p-*value	Model 2	*p-*value	Model 3	*p-*value
**In-hospital mortality**	Q1	Reference	< 0.001; ^***^	Reference	< 0.001; ^***^	Reference	< 0.001; ^***^
	Q2	6.62 (1.48, 29.69)	0.014; ^*^	6.16 (1.37, 27.68)	0.018; ^*^	4.64 (1.02, 21.05)	0.047; ^*^
	Q3	8.76 (2.02, 38.04)	0.04; ^*^	7.65 (1.76, 33.32)	0.007; ^**^	4.51 (1.02, 19.97)	0.047; ^*^
	Q4	41.23 (10.08, 168.62)	< 0.001; ^***^	35.87 (8.75, 147.12)	< 0.001; ^***^	14.98 (3.56, 63.03)	< 0.001; ^***^
**30-day post-admission mortality**	Q1	Reference	< 0.001; ^***^	Reference	< 0.001; ^***^	Reference	< 0.001; ^***^
	Q2	8.78 (2.02, 38.17)	0.004; ^**^	7.87 (1.81, 34.23)	0.006; ^**^	6.42 (1.47, 27.96)	0.013; ^*^
	Q3	14.35 (3.42, 60.24)	< 0.001; ^***^	11.69 (2.78, 49.12)	< 0.001; ^***^	7.99 (1.89, 33.76)	0.005; ^**^
	Q4	51.91 (12.80, 210.50)	< 0.001; ^***^	41.94 (10.33, 170.28)	< 0.001; ^***^	22.53 (5.47, 92.87)	< 0.001; ^***^
**90-day post-admission mortality**	Q1	Reference	< 0.001; ^***^	Reference	< 0.001; ^***^	Reference	< 0.001; ^***^
	Q2	3.61 (1.89, 6.89)	< 0.001; ^***^	3.20 (1.68, 6.12)	< 0.001; ^***^	2.69 (1.41, 5.14)	0.003; ^**^
	Q3	5.86 (3.17, 10.83)	< 0.001; ^***^	4.70 (2.54, 8.69)	< 0.001; ^***^	3.28 (1.76, 6.10)	< 0.001; ^***^
	Q4	16.00 (8.91, 28.73)	< 0.001; ^***^	12.84 (7.14, 23.09)	< 0.001; ^***^	7.18 (3.93, 13.14)	< 0.001; ^***^

Model results are shown as hazard ratios or odds ratios with 95% confidence intervals. Red cell distribution width was grouped as follows: Q1: ≤ 13.5, Q2: 13.5–14.6, Q3: 14.6–16.1, Q4: > 16.1.

Model 1: Categorized red cell distribution width without adjustment.

Model 2: Model 1 adjusted by age, gender, race.

Model 3: Model 2 additionally adjusted for comorbidities (chronic heart failure, myocardial infarction, peripheral vascular disease, cerebrovascular disease, chronic pulmonary disease, rheumatic disease, peptic ulcer disease, paraplegia, diabetes, liver disease, cancer, renal disease) and interventions (mechanical ventilation, hemodialysis and vasopressor administration).

Statistical significance was set at *p* < 0.05.

* *p* < 0.05;

** *p* < 0.01;

*** *p* < 0.001.

### ROC analysis, RCS curves and Kaplan-Meier curves

**Fig 2:**
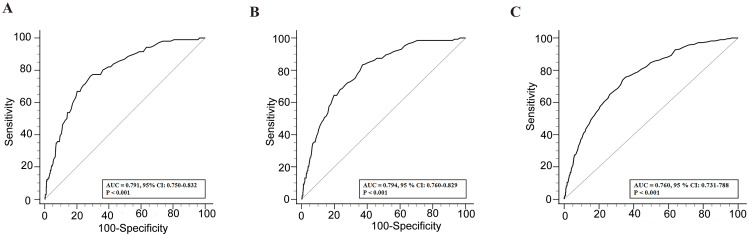
ROC curves for RDW and each all-cause mortality rate. **(A)** In-hospital all-cause mortality; **(B)**. 30-day post-admission all-cause mortality; **(C)**. 90-day post-admission all-cause mortality.

**Fig 3:**
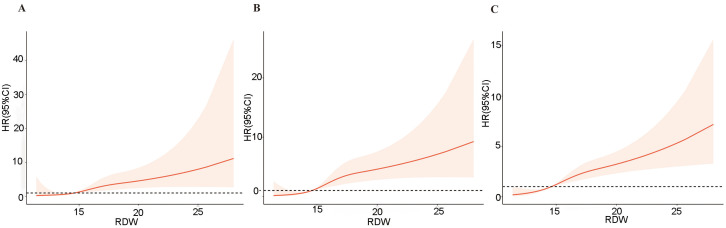
The results of the RCS curve. **(A)** In-hospital all-cause mortality; **(B)**. 30-day post-admission all-cause mortality; **(C)**. 90-day post-admission all-cause mortality.

**Fig 4:**
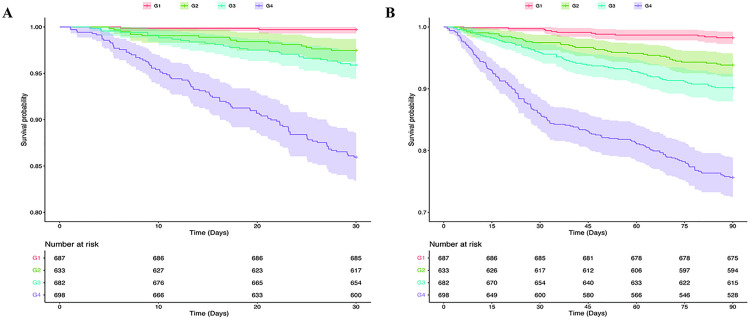
The Kaplan-Meier survival analysis curve. **(A)** The high RDW group; **(B)** The low RDW group.

We plotted the ROC curves for RDW and each all-cause mortality rate (Fig 2), which showed that RDW was significantly effective in predicting in-hospital all-cause mortality (AUC: 0.791, 95% CI: 0.750–0.832), 30-day post-admission all-cause mortality (AUC: 0.794, 95% CI: 0.760–0.829), and 90-day post-admission all-cause mortality (AUC: 0.760, 95%CI: 0.731–0.788). The RCS curve results (Fig 3) indicate a positive association between increasing RDW levels and a higher risk of all-cause mortality during hospitalization, as well as at 30 and 90 days post-admission. The Kaplan-Meier survival curves (Fig 4) showed significantly higher mortality rates in the high RDW group compared to the low RDW group at all observed time points: in-hospital, 30 days post-admission, and 90 days post-admission (*p* < 0.001).

### Subgroup analysis

Table 3 highlights the consistent association between RDW levels and all-cause mortality across different subgroups, including in-hospital, 30-day, and 90-day post-admission periods. When stratified by sex, CHF, diabetes, and myocardial infarction, no significant interaction was observed between RDW and any subgroup (*p*: 0.254–0.920). In conclusion, RDW is an independent prognostic factor.

**Table 3:** Multivariate Cox or logistic regression analyses for categorized RDW and clinical outcomes in patients with osteomyelitis in different subgroups according to the fully adjusted model (Model 3).

Subgroups		Results [HR/OR, (95% CI), *P* value]					
		In-hospital mortality	*p-*value for interaction	30-day post-admission mortality	*p-*value for interaction	90-day post-admission mortality	*p-*value for interaction
**Gender (male)**	Q1	Reference	0.592	Reference	0.915	Reference	0.757
	Q2	4.17 (0.49, 35.80), 0.193		8.96 (1.15, 69.76), 0.036		3.18 (1.37, 7.35), 0.007	
	Q3	5.70 (0.71, 45.47), 0.101		10.25 (1.35, 77.68), 0.024		3.96 (1.76, 8.90), < 0.001	
	Q4	18.74 (2.47, 142.30), 0.005		29.06 (3.93, 214.66), < 0.001		8.19 (3.70, 18.13), < 0.001	
**Gender (female)**	Q1	Reference		Reference		Reference	
	Q2	5.48 (0.64, 47.12), 0.122		4.57 (0.53, 39.36), 0.166		2.42 (0.86, 6.83), 0.094	
	Q3	2.50 (0.28, 22.42), 0.413		5.56 (0.70, 44.18), 0.105		2.60 (0.97, 6.96), 0.058	
	Q4	9.18 (1.18, 71.65), 0.034		17.13 (2.30, 127.68), 0.006		6.61 (2.60, 16.79), < 0.001	
**CHF (-)**	Q1	Reference	0.254	Reference	0.543	Reference	0.872
	Q2	4.41 (0.51, 38.45), 0.179		8.04 (1.02, 63.65), 0.048		2.70 (1.21, 6.00), 0.015	
	Q3	6.51 (0.81, 52.68), 0.079		12.84 (1.70, 97.15), 0.013		3.44 (1.59, 7.48), 0.002	
	Q4	19.68 (2.58, 150.34), 0.004		26.60 (3.58, 197.65), 0.001		7.22 (3.38, 15.42), < 0.001	
**CHF (+)**	Q1	Reference		Reference		Reference	
	Q2	4.10 (0.47, 35.59), 0.200		4.25 (0.52, 34.65), 0.176		2.33 (0.76, 7.10), 0.137	
	Q3	2.04 (0.24, 17.35), 0.512		3.40 (0.44, 26.47), 0.243		2.66 (0.93, 7.58), 0.068	
	Q4	7.26 (0.94, 56.04), 0.057		11.86 (1.63, 86.55), 0.015		6.10 (2.22, 16.73), < 0.001	
**Diabetes (-)**	Q1	Reference	0.823	Reference	0.920	Reference	0.433
	Q2	5.36 (0.63, 45.86), 0.126		6.73 (0.84, 54.17), 0.073		2.57 (1.02, 6.47), 0.046	
	Q3	4.26 (0.50, 35.97), 0.184		7.93 (1.03, 61.30), 0.047		2.89 (1.19, 7.05), 0.019	
	Q4	12.56 (1.62, 97.62), 0.016		22.31 (3.00, 165.70), 0.002		6.10 (2.56, 14.53), < 0.001	
**Diabetes (+)**	Q1	Reference		Reference		Reference	
	Q2	4.41 (0.51, 37.91), 0.177		6.25 (0.78, 50.23), 0.085		2.81 (1.12, 7.02), 0.027	
	Q3	4.15 (0.51, 33.88), 0.184		7.87 (1.03, 60.07), 0.047		3.56 (1.49, 8.51), 0.004	
	Q4	17.07 (2.24, 129.96), 0.006		23.49 (3.19, 172.89), 0.002		8.72 (3.76, 20.26), < 0.001	
**MI (-)**	Q1	Reference	0.357	Reference	0.297	Reference	0.576
	Q2	7.65 (0.96, 61.30), 0.055		9.26 (1.19, 71.91), 0.033		2.53 (1.27, 5.02), 0.008	
	Q3	8.74 (1.13, 67.69), 0.038		14.57 (1.96, 108.57), 0.009		3.22 (1.67, 6.20), < 0.001	
	Q4	25.40 (3.42, 188.71), 0.002		38.29 (5.25, 279.53), < 0.001		6.56 (3.47, 12.41), < 0.001	
**MI (+)**	Q1	Reference		Reference		Reference	
	Q2	1.19 (0.11, 13.47), 0.887		2.07 (0.23, 18.88), 0.518		3.32 (0.40, 27.56), 0.266	
	Q3	1.02 (0.09, 11.23), 0.985		2.26 (0.26, 19.98), 0.462		4.23 (0.54, 33.36), 0.171	
	Q4	4.59 (0.51, 41.63), 0.176		7.64 (0.98, 59.72), 0.053		13.65 (1.82, 102.51), 0.011	

RDW was grouped as follows: Q1: ≤ 13.5, Q2: 13.6–14.6, Q3: 14.7–16.1, Q4: > 16.1.

Model 3: Categorized red cell distribution width adjusted for age, gender, race, and comorbidities and interventions mentioned in the statistical analysis section.

Statistical significance was set at p < 0.05.

HR: Hazard Ratio; OR:Odds Ratio; CI:Confidence Interval; CHF:Congestive Heart Failure; MI:Myocardial Infarction.

## Discussion

To the best of our knowledge, this retrospective observational study is the first to investigate the association between initial-day RDW levels and overall mortality in adult patients with osteomyelitis admitted to the ICU. Our findings suggest that RDW levels may serve as a reliable and independent predictive biomarker for overall mortality in ICU patients with osteomyelitis. This association remained significant even after adjusting for potential confounding factors. Furthermore, our subgroup analysis revealed that RDW levels continued to predict all-cause mortality in ICU patients with osteomyelitis.

RDW is a measure of the degree of heterogeneity in red blood cell (RBC) volume [18]. Li et al. found a significant and independent positive correlation between RDW and inflammatory biomarkers, such as high-sensitivity C-reactive protein (hsCRP) and erythrocyte sedimentation rate (ESR) [21]. Several studies have identified that certain inflammatory cytokines (TNF-α, interleukin-1, IL-6, and interferon-γ [INF-γ]) disrupt the response to erythropoietin, inhibit RBC production and lifespan, resulting in variations in RBC volume and subsequently elevated RDW [22–24]. Furthermore, inflammatory cytokines can impair iron metabolism, leading to irregular RBC size and shape, which contributes to elevated RDW levels [22,25].

Osteomyelitis is an inflammatory condition caused by the invasion of bone tissue by bacterial pathogens, with *Staphylococcus aureus* being the most common causative agent [26]. Among its virulence factors, *Staphylococcus aureus* produces β-hemolysin (Hlb), which reduces red blood cell count, and α-toxin, which induces the production of inflammatory cytokines such as IFN-γ[27]. It can be inferred that in osteomyelitis cases caused by *Staphylococcus aureus*, the resulting inflammatory response alters red blood cell quantity and size, thereby contributing to elevated RDW levels.

Although no previous studies have examined the relationship between RDW levels and all-cause mortality in osteomyelitis, associations between RDW and all-cause mortality have been reported in other diseases. In other bone-related diseases, Wang et al. reported that elevated RDW levels were significantly associated with mortality in elderly patients with hip fracture (HR: 1.03, 95% CI: 1.02 to 1.05, **p* *< 0.0001, after adjustment) [28]. Similarly, a meta-analysis by Zhu et al. showed that RDW could serve as a predictor of mortality following hip fracture (HR: 3.14, 95% CI: 1.38 to 7.14, **p* *< 0.001) [29]. In the ICU setting, Deniz et al. demonstrated that elevated RDW levels (> 16.5%) were significantly associated with all-cause mortality in patients (OR: 3.27, 95% CI: 2.58 to 4.14, **p* *< 0.001, after adjustment) [30]. This study fills a gap in the literature by demonstrating that RDW is a significant predictor of all-cause mortality in ICU patients with osteomyelitis.

The main strength of our study is that it demonstrates elevated RDW levels as a strong, independent predictor of all-cause mortality in critically ill patients with osteomyelitis. However, this study also has several limitations. Firstly, this was a retrospective study, and therefore, retrospective bias could not be avoided. Secondly, although we adjusted for a number of confounding variables and performed subgroup analyses, unmeasured confounders may still have influenced the results. Thirdly, this was a single-centre study, and thus, further rigorous prospective studies are needed to validate our conclusion. Fourthly, our study population was predominantly White, so whether these findings apply to other ethnic groups require further investigations. Fifthly, due to the limitation in the available literature and data, the relationship between anemia and RDW remains unclear, and further research is needed to explore this association. Given that RDW is commonly elevated in anemic states and that the Q4 group (with higher RDW) also exhibited lower hematocrit and hemoglobin levels along with a higher mean age, the presence of anemia in this subgroup is plausible. The lack of adjustment for anemia may have influenced the observed association between RDW and mortality. Future studies should incorporate anemia status as a covariate to further elucidate the independent prognostic value of RDW in this population. Finally, the variability of RDW cannot be definitively determined; it may fluctuate over time, and its stability remains uncertain. [31] Although current data indicate an association between RDW and osteomyelitis, further research is needed to clarify this relationship.

## Conclusion

A significant association between RDW and increased all-cause mortality has been observed in patients with osteomyelitis. These findings suggest that RDW may serve as a useful marker for identifying individuals at higher risk of mortality during hospitalization. However, further prospective studies are required to clarify the causal relationship and validate the clinical utility of RDW in this population.

## Supporting information

S1 Table The original data for analysis. (XLSX)
